# NTRK2 expression in gastrointestinal stromal tumors with a special emphasis on the clinicopathological and prognostic impacts

**DOI:** 10.1038/s41598-024-51211-7

**Published:** 2024-01-08

**Authors:** Keita Sasa, Raku Son, Akiko Oguchi, Karin Ashizawa, Nobuhiko Hasegawa, Daisuke Kubota, Yoshiyuki Suehara, Tatsuya Takagi, Taketo Okubo, Keisuke Akaike, Kiichi Sugimoto, Makoto Takahashi, Kazuhiro Sakamoto, Takashi Hashimoto, Shinji Mine, Tetsu Fukunaga, Muneaki Ishijima, Takuo Hayashi, Takashi Yao, Yasuhiro Murakawa, Tsuyoshi Saito

**Affiliations:** 1 Department of Human Pathology, Juntendo University School of Medicine, Tokyo, Japan; 2 Department of Medicine for Orthopaedics and Motor Organ, Juntendo University School of Medicine, Tokyo, Japan; 3 RIKEN Center for Integrative Medical Sciences, Yokohama, Japan; 4 Department of Orthopaedic Surgery, Yamanashi Central Hospital, Yamanashi, Japan; 5 Department of Coloproctological Surgery, Graduate School of Medicine, Juntendo University, Tokyo, 113-8421 Japan; 6 Department of Upper Gastroenterological Surgery, Juntendo University Hospital, Bunkyo-ku, Tokyo, 113-8431 Japan; 7 Intractable Disease Research Center, Graduate School of Medicine, Juntendo University, Tokyo, 113-8421 Japan; 8 Institute for the Advanced Study of Human Biology (ASHBi), Kyoto University, Kyoto, Japan; 9 IFOM ETS - the AIRC Institute of Molecular Oncology, Milan, Italy

**Keywords:** Cancer, Oncology, Pathogenesis

## Abstract

Gastrointestinal stromal tumors (GISTs) are typically characterized by activating mutations of the KIT proto-oncogene receptor tyrosine kinase (*KIT*) or platelet-derived growth factor receptor alpha (*PDGFRA*). Recently, the neurotrophic tyrosine receptor kinase (*NTRK*) fusion was reported in a small subset of wild-type GIST. We examined trk IHC and *NTRK* gene expressions in GIST. Pan-trk immunohistochemistry (IHC) was positive in 25 (all 16 duodenal and 9 out of 16 small intestinal GISTs) of 139 cases, and all pan-trk positive cases showed diffuse and strong expression of c-kit. Interestingly, all of these cases showed only trkB but not trkA/trkC expression. Cap analysis of gene expression (CAGE) analysis identified increased number of genes whose promoters were activated in pan-trk/trkB positive GISTs. Imbalanced expression of *NTRK2*, which suggests the presence of *NTRK2* fusion, was not observed in any of trkB positive GISTs, despite higher mRNA expression. TrkB expression was found in duodenal GISTs and more than half of small intestinal GISTs, and this subset of cases showed poor prognosis. However, there was not clear difference in clinical outcomes according to the trkB expression status in small intestinal GISTs. These findings may provide a possible hypothesis for trkB overexpression contributing to the tumorigenesis and aggressive clinical outcome in GISTs of duodenal origin.

## Introduction

Gastrointestinal stromal tumor (GIST) is the most common soft tissue sarcoma of the digestive tract, with a worldwide prevalence of 10–15 cases per million^1–3^. The median age at diagnosis is approximately 60 years, and cases are approximately evenly distributed between sexes^1^. GIST has been reported to originate from the interstitial cells of Cajal. As intermediates between the autonomic nervous system and smooth muscle cells in the gastrointestinal tract, these cells are involved in the regulation of motility and autonomic function^4,5^. GISTs arise predominantly in the stomach (55.6%) and small intestine (31.8%), with the rectum (6%), esophagus (0.7%), and various other locations (5.5%) accounting for the remaining cases^1^.

Diagnosis is based on histological features in addition to the clinical course and tumor location. GISTs also have distinct molecular characteristics, such as immunohistochemical expression of DOG-1^6^. Most cases are characterized by activating mutations of the *KIT* proto-oncogene receptor tyrosine kinase (*KIT*, 75–80%) or platelet-derived growth factor receptor alpha (*PDGFRA*, 10%)^3^.

Surgery is the initial treatment for primary and localized cases, and drug therapy is the second-line treatment for more advanced cases^7^. Imatinib is a selective tyrosine kinase inhibitor (TKI) and contributes to improving the prognosis of advanced GISTs^8,9^. However, due to the poor efficacy of imatinib in some cases involving *PDGFRA* mutations and *KIT* and *PDGFRA* wild-type GISTs, it is necessary to develop new targets for therapy^3^. Recently, the existence of the neurotrophic tyrosine receptor kinase (*NTRK*) fusion gene has been reported in a small subset of wild type GISTs, suggesting the effectiveness of a new TKI in this tumor^10–12^. However, there are no other reports, and controversy remains^13^. In this study, we analyzed NTRK expression and its clinical significance in GISTs.

## Materials and methods

### Case selection

We examined 139 cases of GIST. Prognostic information was collected from files in the Department of Human Pathology, Juntendo University Hospital, Tokyo, Japan. All patients were treated at the Juntendo University Hospital between 2008 and 2020. These cases were diagnosed using the WHO classification system for soft-tissue tumors and classified using the modified Fletcher classification^14^. Diagnoses were confirmed by immunohistochemical analysis of DOG1 and c-kit expression. Clinicopathologic data of the 139 patients are shown in Table 1. The follow-up periods ranged from 0.1 to 182 months (mean: 61.3 months). Patients were treated with surgical resection without a pre-adjuvant treatment, such as that with imatinib. In 137 cases, the tumors were completely resected. Tissue microarray (TMA) blocks, each consisting of 2 mm cores, were made for these cases. All experiments were performed in accordance with relevant guidelines and regulations of the institution and the Declaration of Helsinki.

**Table 1:** Clinicopathological data in 139 GIST cases.

	n = 139	P-value
Age average	66.19 (33–88)	0.9756
< 60	33	
60–69	50	
≧70	56	
Sex		0.5951
Female	61	
Male	78	
Primary site		< 0.0001
Esophagus	3	
Stomach	103	
Duodenum	16	
Small intestine	16	
Other	1	
Size (mm)		0.0054
≦ 20	19	
21–50	65	
51–100	45	
> 100	10	
Mitotic figure		< 0.0001
≦ 5	109	
6–10	16	
> 10	14	
Modified fletcher classification		< 0.0001
Very low	17	
Low	58	
Intermediate	29	
High	35	
MIB-1 index (%)		< 0.0001
< 10	107	
10–29	22	
≧ 30	10	

### Immunohistochemistry (IHC)

IHC staining was performed for all cases using the antibodies described in Supplementary Table 1. The expression of tropomyosin receptor kinase (TRK) A, trk B, and trk C encoded by either *NTRK1, NTRK2 or NTRK3* was examined by IHC.

### RNA extraction

For NanoString analysis, RNA was extracted from formalin-fixed paraffin-embedded (FFPE) samples using the RNeasy FFPE Kit (QIAGEN, Hilden, Germany). For CAGE analysis, RNA was extracted from fresh-frozen samples using the RNeasy Plus Mini Kit (QIAGEN, Hilden, Germany). RNA concentration was measured using Nanodrop2000 (Thermo Fisher Scientific, Inc. MA).

### DNA extraction

DNA was extracted from tumoral and corresponding non-tumoral tissues using the QIAamp DNA FFPE Tissue Kit (QIAGEN, Hilden, Germany) according to the manufacturer’s protocols. Samples were treated with RNase A accordingly, and DNA concentration was measured using Nanodrop2000.

### Nanostring-based mRNA imbalance analysis

Nanostring (NanoString Technologies, Inc., Seattle, WA, USA) analysis was performed (probe set described in Supplementary Table 2) to target a total of 32 genes as previously described^15^. This assay can estimate the formation of fusion genes by comparing the mRNA expression levels at the 5ʹ-side and 3ʹ-side. Briefly, 250–400 ng of ribonucleic acid (RNA) was hybridized to the probes (a reporter probe and a capture probe) at 65 °C for 18–24 h using a thermal cycler. Samples were added into the nCounter Prep Station for 3 h to remove excess probes, purify, and immobilize the sample on the internal surface of the cartridge. Finally, the sample cartridge was transferred to the nCounter Digital Analyzer, where color codes were counted and tabulated for each target molecule. The expression number for the base sequence of the probe part was analyzed using nSolver Analysis Software Version 4.0 (https://www.nanostring.com/products/analysis-software/nsolver).

### NanoString-based copy number variation (CNV) analysis

NanoString-based CNV analysis was performed using nCounter (NanoString Technologies, Seattle, WA, USA) as previously described^16^. The probe list used in this assay is described in Supplementary Table 3. Three probes were prepared for each of the total 24 genes. According to the sample status, 150–300 ng of DNA was processed for the NanoString nCounter CNV analysis according to the manufacturer’s protocol (NanoString Technologies, Seattle, WA, USA). The CNV for the base sequence of the probe part was analyzed using nSolver Analysis Software Version 4.0. For GIST clinical samples, each data point was normalized by dividing each score from the tumoral DNA by the score from the corresponding non-tumoral DNA. The mean of the three normalized scores for each gene was then calculated. The cut-off for amplification was defined as 2.0.

### Quantitative polymerase chain reaction for trk ligands

Each trk has specific ligands. Various neurotrophins (NT), including NT-4 (NT-5), NT-3, nerve growth factor (NGF), and brain-derived neurotrophic factor (BDNF) have been reported as ligands. BDNF, NT-4 (NT-5), and NT-3 are known to bind to trkB and activate downstream trkB signaling^17,18^. We examined the expression of ligands BDNF, NT-4, and NT-3 using qPCR. All quantitative real-time PCR (qPCR) was performed with TaqMan Fast Advanced Master Mix (Applied Biosystems) on an Applied Biosystems Step One Plus Real Time PCR System in accordance with standard protocols. qPCR was performed using predeveloped TaqMan assays (20× Primer Probe mix; Applied Biosystems, CA, USA) for BDNF (Assay ID Hs02718934_s1), NT3 (Assay ID Hs00267375_s1), NT4 (Assay ID Hs01921834_s1), and GAPDH (Assay ID Hs02786624_g1). The amount of each target gene relative to the *GAPDH* housekeeping gene was determined using the comparative threshold cycle (Ct) method.

### CAGE analysis protocol

We analyzed the promoter activity profiles in 10 GISTs, using the CAGE protocol. The 10 cases consisted of eight gastric, one duodenal, and one small intestinal GISTs, with the duodenal and small intestinal GIST being trkB-positive. CAGE libraries were prepared and sequenced in K.K.DNAFORM. The reads were mapped to the reference genome (GRCh38) by STAR v2.7.10^19^. The aligned reads were counted on regions of GENCODE transcription start sites ± 300 base pairs (GENCODE v41). Count data were normalized as counts per million by edgeR v3.34.0 and the subsequent analysis was performed by R v4.1.0.

### KIT mutational analysis for GISTs with pan-trk/trkB expression

Information of genotype of GIST cases were obtained from each medical record where available. *KIT* mutational analysis was performed for remaining GIST cases with pan-trk/trkB expression as described previously^20^. Several cases were excluded from this analysis due to the short of materials or inadequate sample quality.

### Statistical analysis

Categorical variables were analyzed using Fisher’s exact or chi-square test. Column variable was analyzed using the Mann–Whitney test. To determine prognosis, Kaplan–Meier survival analysis was performed. The date of surgical resection was set as the starting point and the date of death, date of recurrence, or last date of follow-up was used as the end point. Statistical analyses were performed using GraphPad Prism® software version 9.4.0 (GraphPad, San Diego, CA, USA). p value of < 0.05 was considered statistically significant.

### Ethical standards

This study was reviewed and approved by the Juntendo University School of Medicine Institutional Review Board (#21-079). The informed consents were obtained from all subjects and/or their legal guardian(s).

## Results

### Clinicopathological analysis

**Figure 1:**
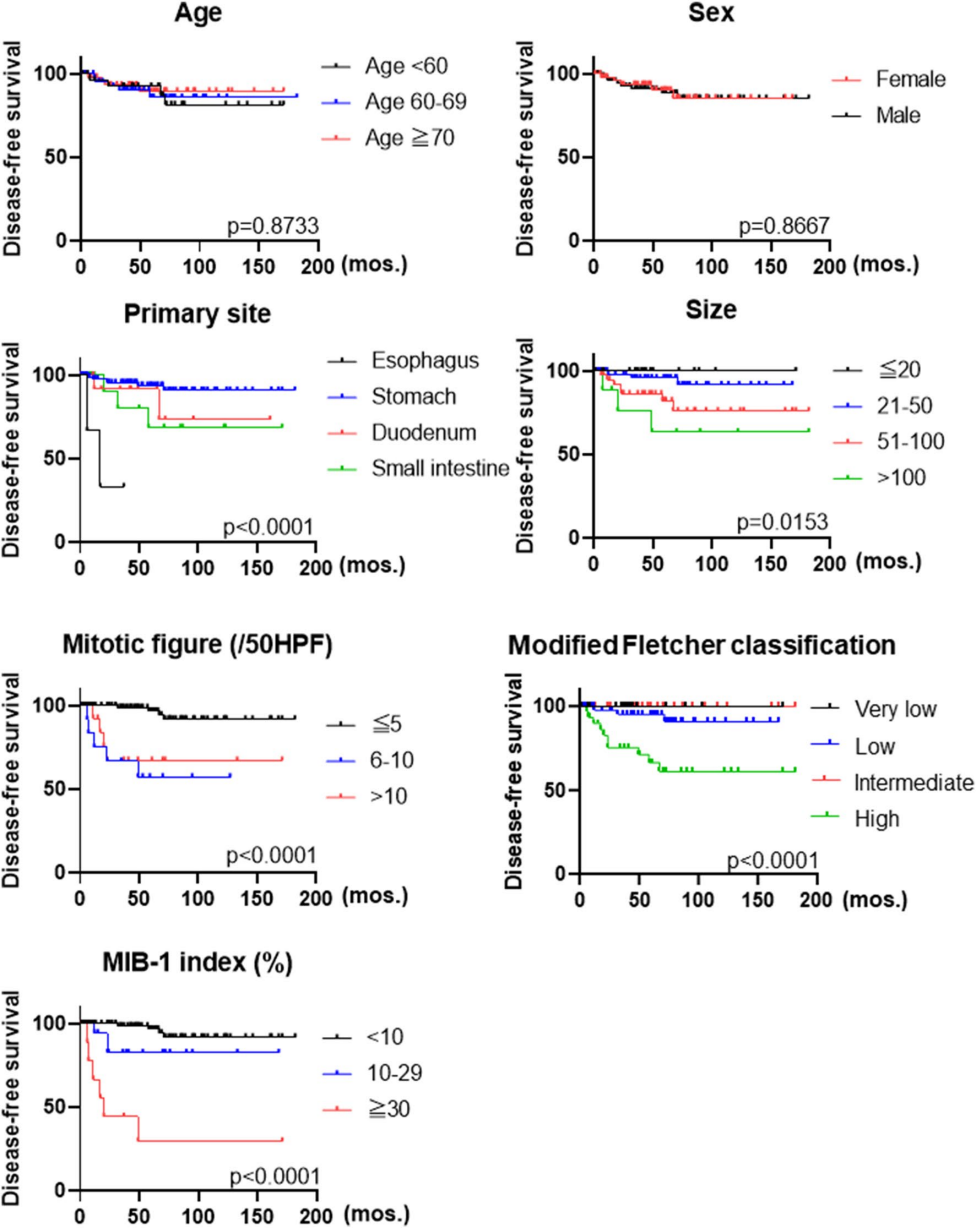
Survival analysis by clinicopathological parameters in this series of GISTs. Duodenal GISTs show statistically significant shorter disease-free survival. Additionally, higher mitotic figures, risk-classification, and MIB-1 LI affect poor prognosis with statistical significance.

Clinicopathologic characteristics of the 139 patients are summarized in Table 1. Briefly, there were 3 esophageal GISTs (2%), 103 gastric GISTs (74%), 16 duodenal GISTs (11.5%), 16 small intestinal GISTs (11.5%), and 1 vaginal case. Seventeen cases (12%) were classified as very low risk, 58 cases (42%) as low risk, 29 cases (21%) as intermediate risk, and 35 cases (25%) as high risk. Primary site (p < 0.0001), larger tumor size (p = 0.0153), higher mitotic figure (p < 0.0001), higher modified Fletcher classification (0 < 0.0001) and higher MIB-1 index (p < 0.0001) were significantly associated with poor prognosis. Interestingly, the duodenal GIST showed poor prognosis (p < 0.0001) than other GISTs (Fig. 1). Age and sex were not associated with prognosis.

### Pan-trk expression in GIST

**Figure 2:**
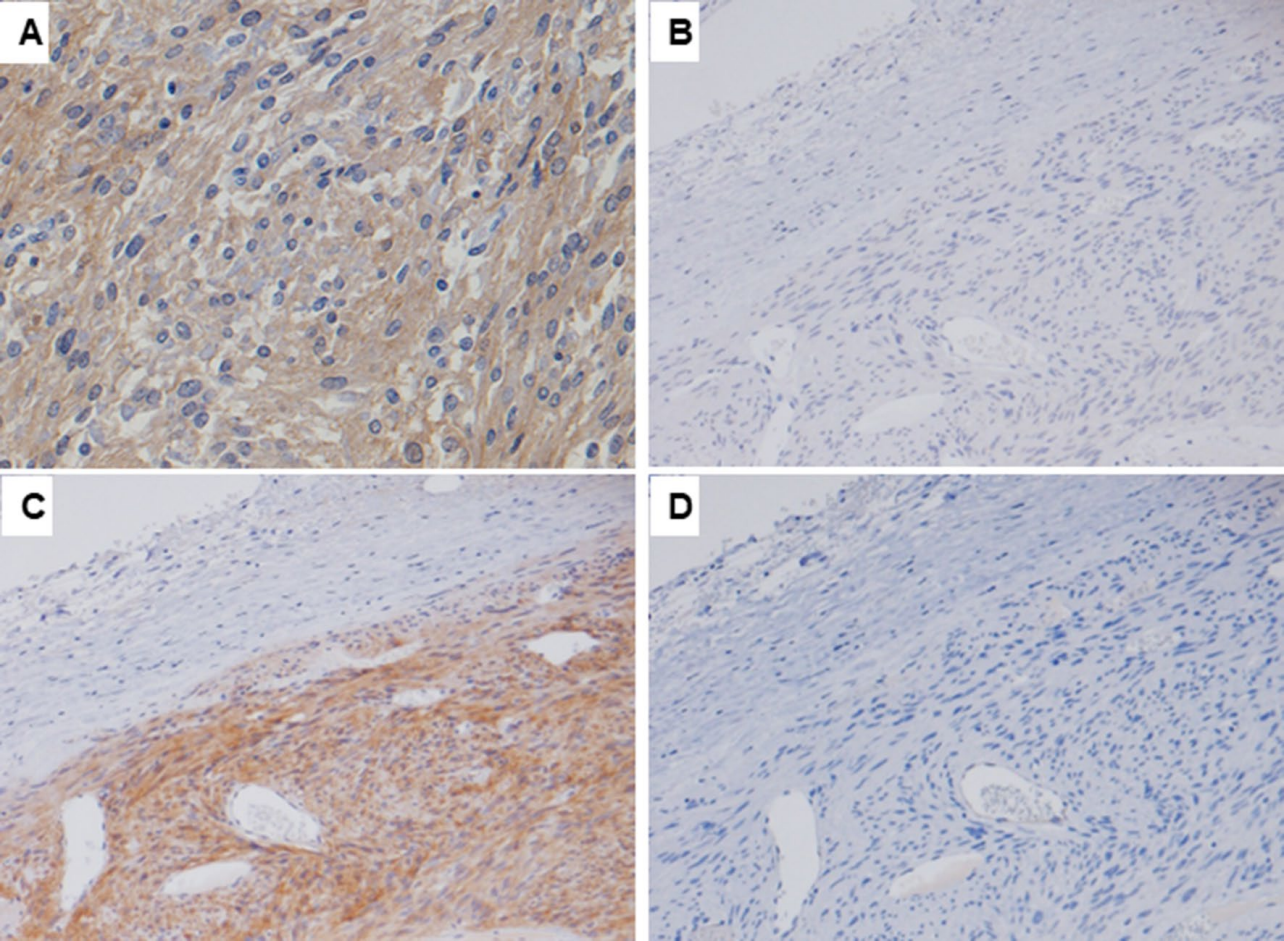
Pan-trk IHC staining shows diffuse and strong expression in a case of GIST (**A**). This case also shows diffuse and strong expression of trkB (**C**), however, it was negative for trkA (**B**) and trkC (**D**).

TMA-based pan-trk IHC identified positively staining in 25 out of 139 GIST cases (Fig. 2A, Table 2). Pan-trk IHC showed diffuse cytoplasmic and membranous staining (Fig. 2A). Furthermore, all pan-trk IHC positive GISTs showed only trkB expression encoded by *NTRK2,* however, trkA and trkC expression was not observed (Fig. 2B–D).

**Table 2:** Clinicopathological characteristics of pan-trk IHC positive GIST.

	Pan-trk IHC (−)	Pan-trk IHC (+)	P-value
	n = 114	n = 25	
Age	67.50 (41–88)	64.00 (33–80)	0.0609
Sex			0.6628
Female	49	12	
Male	65	13	
Primary site			< 0.0001
Esophagus	3	0	
Stomach	103	0	
Duodenum	0	16	
Small intestine	7	9	
Vagina	1	0	
Size (mm)			0.3738
≦ 20	13	6	
21–50	54	11	
51–100	39	6	
> 100	8	2	
Mitotic figure			0.1895
≦ 5	86	23	
6–10	15	1	
> 10	13	1	
Modified fletcher classification			0.6583
Very low/low	60	15	
Intermediate/high	54	10	
MIB-1 index (%)			0.432
< 10	85	22	
10–29	20	2	
≧ 30	9	1	
Imatinib treatment	n = 26	n = 8	0.1456
Response	19	3	
Non response	3	3	
Unknown	4	2	

### Clinicopathological characteristics of pan-trk positive GISTs

**Figure 3:**
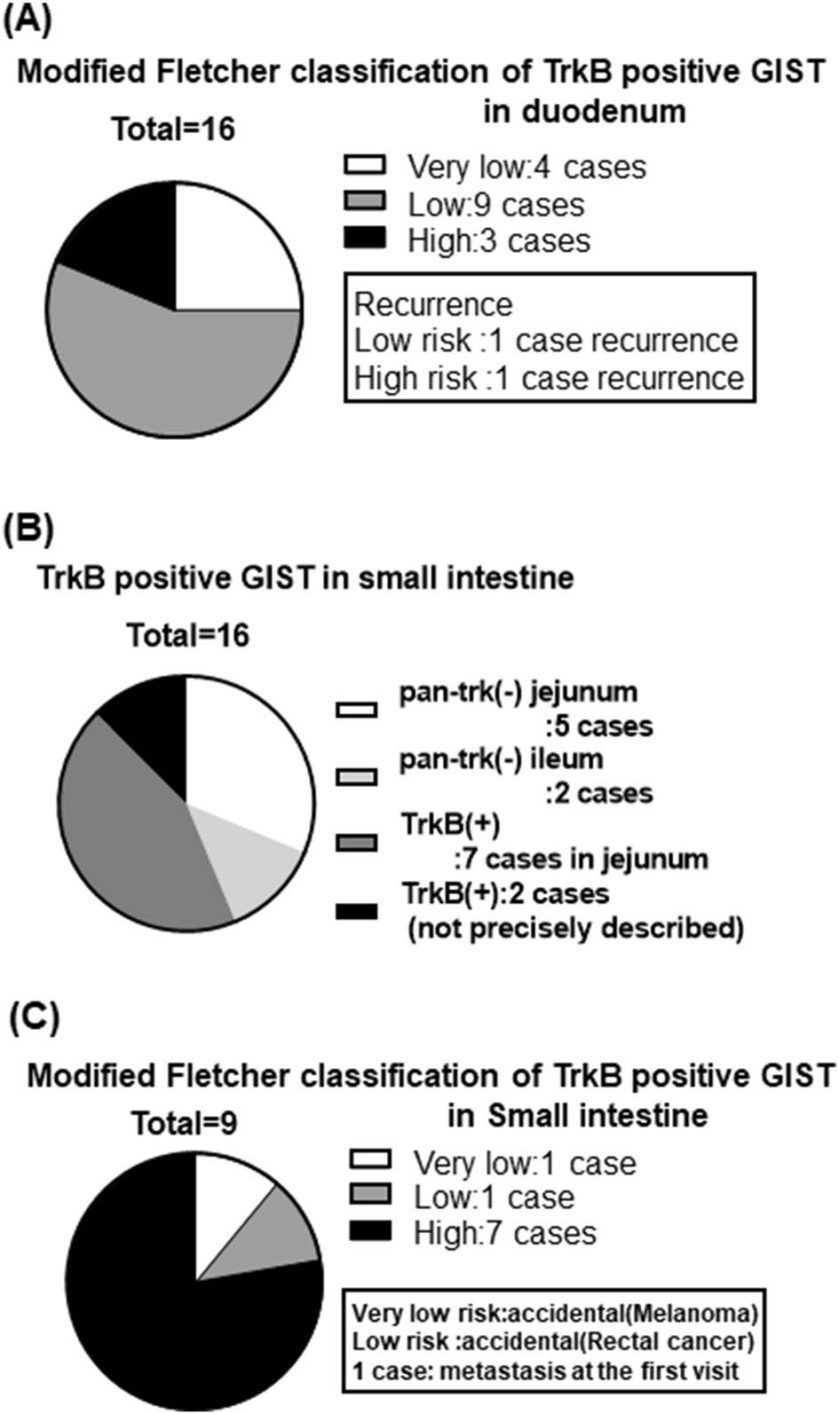
Risk classification of trkB positive GISTs. In duodenal GISTs, 13 out of 16 cases are classified as either very low- or low-risk by modified Fletcher classification (**A**). In small intestinal GISTs, at least 7 out of 12 jejunal GISTs show trkB expression (**B**). In contrast, 7 out of 9 small intestinal GISTs with trkB expression are classified as high-grade (**C**).

**Figure 4:**
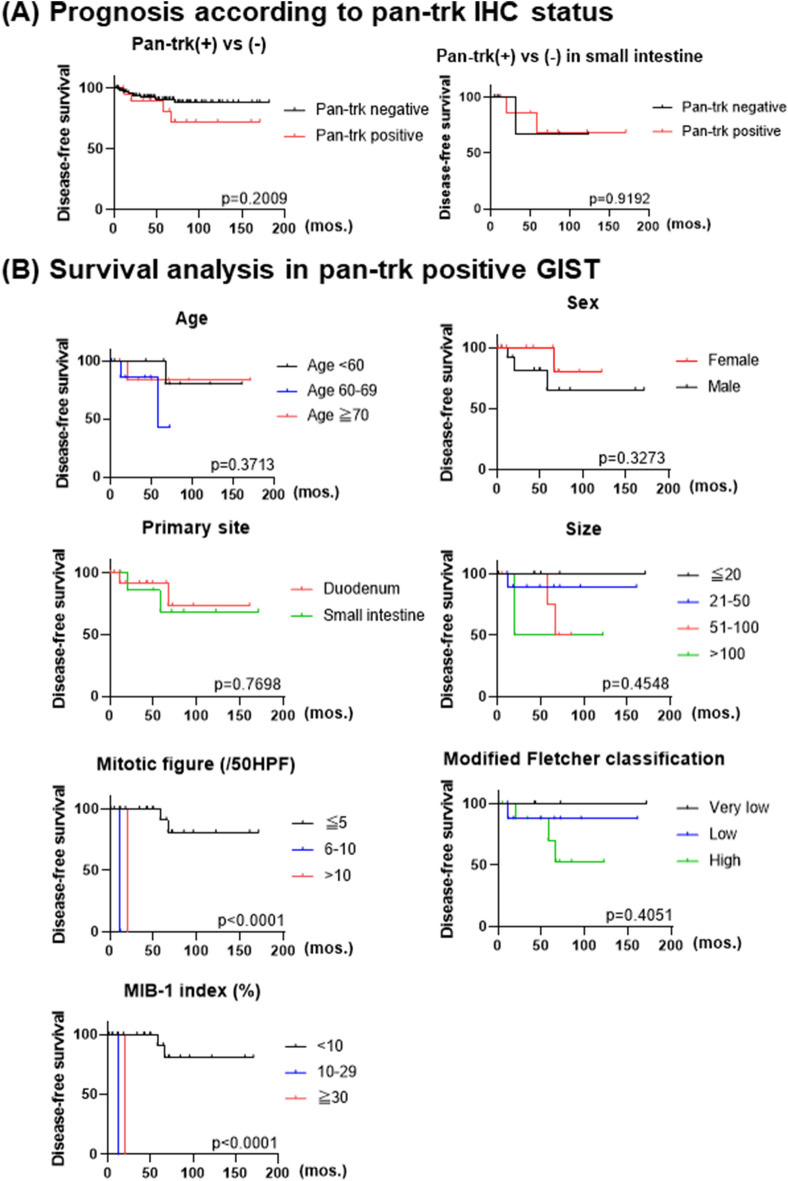
Disease-free survival rate is inferior in pan-trk/trkB positive-GISTs compared with that in pan-trk/trkB negative-cases, although this was not statistically significant (**A**, left). This trend was not clear in small intestinal cases (**A**, right). Survival analysis in pan-trk/trkB positive-GIST reveals that disease-free survival was affected only by mitotic activity and MIB-1 LI (**B**).

Clinicopathological characteristics of pan-trk positive GISTs are summarized in Table 2. Of the primary sites for tumors with pan-trk expression, 16 were duodenal and nine were small intestinal. Interestingly, all of the duodenal cases and more than half of the small intestinal cases (at least seven cases were of jejunal origin) showed pan-trk positive staining (p < 0.0001, Table 2). In addition, all pan-trk positive cases showed diffuse and strong expression of c-kit and harbored *KIT* mutations where information was available. None of pan-trk-positive cases had clinical signs of type 1 neurofibromatosis. By risk classification, 13 out of 16 duodenal GISTs showed very low- or low-risk groups, and recurrence was observed in one case each from low- and high-risk groups. On the other hand, seven out of nine small intestinal GISTs with pan-trk expression were high-risk, and the remaining two cases were discovered incidentally on histological examination of other surgically resected malignant tumors. No recurrence was observed for the small intestinal GISTs with pan-trk expression, despite of their High-grade natures (Fig. 3). Pan-trk IHC-positive GISTs tended to have a poor prognosis, although there was no statistically significant difference between pan-trk IHC-positive GISTs and IHC-negative GISTs (Fig. 4A). None of the patients with pan-trk positive GIST experienced recurrence. In pan-trk IHC-positive GISTs, primary site, age, sex, modified Fletcher classification, and size were not significantly associated with poor prognosis (Fig. 4B), whereas mitotic figure (p < 0.0001) and MIB-1 index (p < 0.0001) were significantly associated with poor prognosis (Fig. 4B).

### NanoString assay for the pan-trk IHC positive GISTs

**Figure 5:**
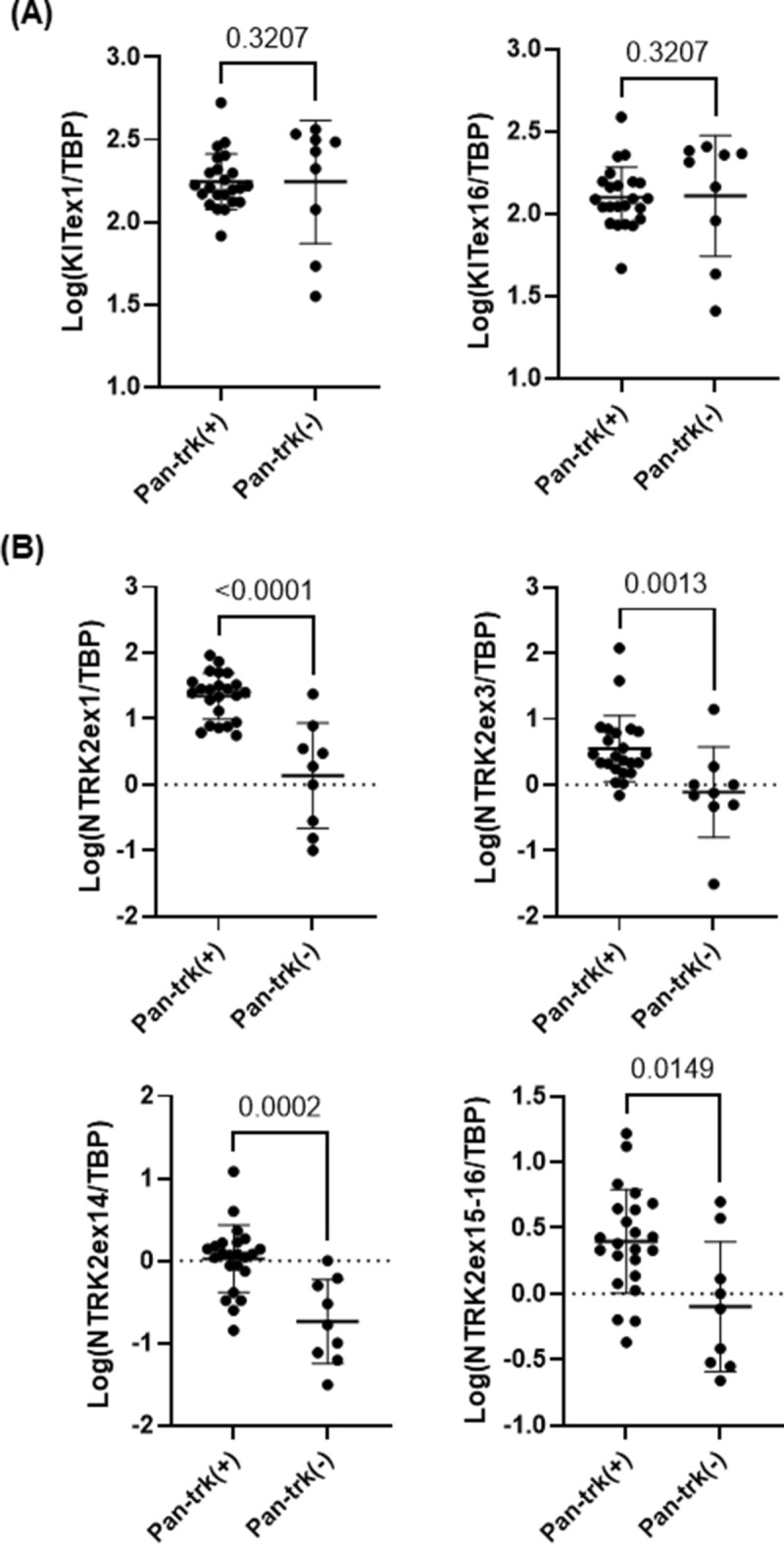
*KIT* expression level did not differ according to the trk IHC status (**A**). Both 5ʹ-side and 3ʹ-side expressions of *NTRK2* mRNA are significantly higher in GIST samples with trkB expression than in those without (**B**).

Next, to examine whether trkB expression reflected the presence of *NTRK2* fusion, we performed NanoString imbalance assay for 23 out of 25 cases with trkB expression in 30 tyrosine kinase genes including *NTRK1-3*^15,21,22^. Two cases were not available for adequate tissue for this analysis. Imbalanced expression of *NTRK1-3* was not observed in any of the analyzed GISTs (Supplementary Fig. 1, Supplementary Table 4). Interestingly, NanoString-based mRNA imbalance analysis revealed constantly high expression of *KIT* in all the cases with trkB expression, in line with the c-kit IHC findings. High expression of *NTRK2* was observed in all the cases with trkB expression, but not for *NTRK1* and *NTRK3* (Supplementary Table 5, Fig. 5). NanoString-based CNV analysis revealed that one case (Case#76) showed *NTRK1* amplification (× 2.3; 4.6 copies), but no case showed *NTRK2* amplification (Supplementary Table 6). These findings suggested that high expression of *NTRK2* in GIST with trkB expression could be due to transcriptional activation of *NTRK2*.

### Ligand expression of NTRK2

There was no difference in the expression of examined ligands between trkB positive GISTs and trkB negative GISTs. Furthermore, NT-4, the major *NTRK2* ligand, was not expressed even in trkB positive GISTs (Supplementary Fig. 2).

### Profiles of genome-wide promoter activities in GIST

**Figure 6:**
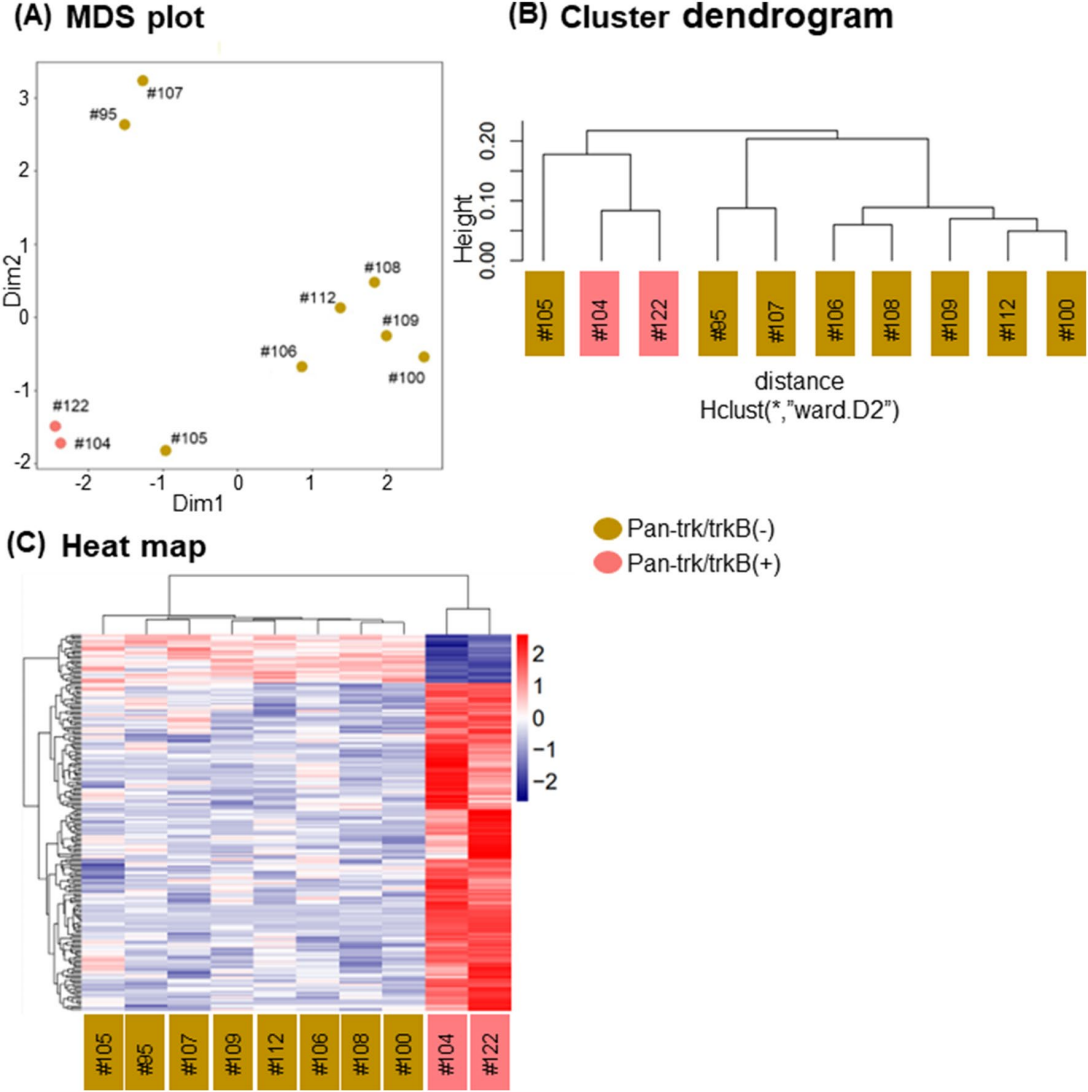
MDS plot and cluster dendrogram reveals that GIST samples with trkB expression (Cases#104, #122) were separately clustered (**A,B**). Furthermore, GIST samples without trkB expression are subdivided according to pfetin expression (**A,B**). Cases#95 and #107 are pfetin-negative cases. GISTs are clearly separated by differentially expressed genes according to trkB expression. The number of genes with promoter activation is higher in GIST samples with trkB expression than in those without (**C**).

We analyzed the promoter activity profiles in eight gastric, one duodenal, and one small intestinal GISTs. This analysis clustered 10 GISTs into three groups according to Pfetin and pan-trk/trkB IHC status (Fig. 6A,B). Pfetin, found to be expressed in approximately 80% of the GISTs, is reported to be a prognostic factor in GIST, and Pfetin negative cases show a poor prognosis^23^. GIST#104 and #122 were of duodenal and small intestinal origin and were positive for pan-trk/trkB and pfetin. GIST#95 and #107 were of gastric origin and were characterized by negative staining for Pfetin/pan-trk/trkB. GIST#100, #105, #106, #108, #109 and #112 were of primary gastric GISTs and were positive for Pfetin and negative for pan-trk/trkB.

The heatmap with 208 differentially expressed promoter (FDR < 0.05) showed different promoter activation patterns between pan-trk positive and negative GISTs (Fig. 6C). Interestingly, mRNA level of trkB ligands by qPCR analysis showed almost the same trend when they were examined in detail using RNA extracted from FFPE samples, although mRNA expression level of *NTRK* by Nanostring analysis was significantly higher in pan-trk/trkB positive group (Fig. 5, Supplementary Fig. 2) Furthermore, CNV analysis did not reveal amplification of *NTRK2* in any of GIST cases (Supplementary Table 6). The promoter activity of *NTRK2* was higher in pan-trk/trkB positive group, compared to pan-trk negative group, although this difference was not statistically significant (FDR = 0.15, p = 0.0067; Not shown). On the other hand, regardless of pan-trk IHC results, there was no difference in the promoter activity of *KIT* (Not shown) as well as *KIT* mRNA expression (Fig. 5). In the promoter activity profiles, as with mRNA expression, there was no difference in the ligand expressions between trkB positive GISTs and trkB negative GISTs (Supplementary Fig. 2). Interestingly, vascular developments including VEGFA-VEGFR2 signaling pathway appeared as one of the differentially activated pathways in pan-trk/trkB positive group by CAGE analysis (Supplementary Fig. 3).

## Discussion

The *NTRK* gene encodes the neurotrophic-tropomyosin receptor tyrosine kinase, and *NTRK1*, *NTRK2*, *NTRK3* encode trkA, trkB, and trkC proteins, respectively. *NTRKs* are involved in the survival and proliferation of nerve cells^24,25^. Several molecules have been reported as trk ligands, including NGF, BDNF, NT-3 and NT-4 (NT-5). When bound, the ligands directly activate downstream effectors of trks^17^. In oncology, it has been reported that this induces tumorigenesis, including differentiation, growth, and apoptosis, thereby showing potential as therapeutic targets in malignant tumors across systemic organs^26,27^. *NTRK*-fusions are known to be the most effective therapeutic targets among tyrosine kinase fusions. Selective TRK inhibitors show high antitumor effects on tumors carrying the *NTRK* fusion^28^. Although *NTRK* fusion is generally quite rare, being detected in less than 1% of cancers, it is reported to occur frequently in a small subset of cancers such as secretory carcinoma of the parotid gland and infantile fibrosarcoma^17^. Furthermore, the significance of *NTRK* amplification or trk overexpression on cancer progression remains unclear. Recently, overexpression of *NTRK1* is shown in 20% of breast cancers, and its involvement in tumorigenesis and susceptibility to selective TRK inhibitors is reported^29^. Thus, it is increasingly important to find *NTRK*/trk alterations other than *NTRK* fusions.

The relationship between GIST and *NTRK* fusion/expression remains controversial, with few reports still scattered. Wang et al. reported that the expression of the *NTRK*-like family member 3 (*SLITRK3*), a member of the Slitrk family of structurally related transmembrane proteins that are involved in controlling neurite outgrowth, is associated with malignancy including recurrence and metastasis of GISTs^30^. Recently, *ETV6-NTRK3* fusion has been reported within a subset of wild-type GISTs^10,11^. Furthermore, Shi, E et al. report the efficacy of TRK inhibitors for this type of GISTs^11^. However, *NTRK* fusion genes are almost always restricted to gastrointestinal mesenchymal tumors other than GISTs, characterized by the lack of DOG1, with c-kit and *NTRK* fusion tumors being distinct^13^. In this study, duodenal and some small intestinal GISTs showed trkB expression encoded by *NTRK2,* at both the mRNA and protein levels. However, Nanostring analysis did not show imbalanced expression of *NTRK2* in any of these trkB positive GISTs, suggesting the absence of *NTRK2* fusion in these tumors. This finding is consistent with strong c-kit expression and *KIT* mutations in these GISTs (Supplementary Table 5), since the reported *NTRK* fusion in GISTs is restricted to wild-type cases^10,11^.

TrkB is involved in formation and maintenance within the nervous system and construction of normal lung tissue^31,32^. The association between neuroblastoma and trkB expression is well known, and its expression is observed in about 30% of these cases^33^. Furthermore, it has been shown that trkB contributes to the growth and differentiation of neuroblastoma cells, suggesting a relationship to a poor prognosis^33,34^. In addition, high expression of trkB has been reported as a poor prognostic factor in cancers of the digestive system, ovaries, prostate, and lungs^33,35^.

In GISTs, tumor size, mitotic rate and tumor location have been reported to be associated with recurrence^36^. Additionally, duodenal GISTs should be considered for aggressive treatment because of their poor prognosis compared with those of other primary sites^37^. In this study, all of the duodenal GISTs showed overexpression of trkB. Molecular genetic characteristics that associate aggressive behavior in duodenal GISTs are still unknown. Our findings may provide a possible hypothesis that trkB overexpression contributes to the tumorigenesis or aggressive clinical outcome in GISTs of duodenal origin. In contrast, more than half of the GISTs of small intestinal origin also showed overexpression of trkB, however, small intestinal GISTs with trkB overexpression did not show clinical disadvantage compared to those without. This point needs to be further evaluated in the future.

CAGE and subsequent MDS plots, cluster dendrograms and heatmaps analysis revealed that GIST samples with trkB expression were separately clustered from those without (Fig. 6A,B). Furthermore, GIST samples without trkB expression were subdivided according to pfetin expression (Fig. 6A,B). Pfetin has been shown to be expressed in approximately 80% of GISTs and to be a favorable prognostic factor in GIST^23^. In addition, our analysis also showed significant differences in gene promoter activity according to pan-trk/trkB status. The number of genes with promoter activation was higher in GIST samples with trkB expression than in those without (Fig. 6C). These findings suggest that the GISTs with trkB expression may have different characteristics from those without. On the other hand, although the presence of ligand is usually required for the activation of the downstream pathways of trk^17,38^, in this study we could not find any difference in the expression of trkB ligands between pan-trk positive and negative GISTs. A recent study demonstrated that overexpression of trkA in breast carcinoma cells led to growth factor-independent proliferation^29^. Regarding this point, we found activation of VEGFA/VEGFR2 signaling pathway in pan-trk/trkB positive GIST by CAGE analysis. Interestingly, microvessel density and vascular endothelial growth factor expression have been shown as adverse prognostic factors^39^. However, trkB involvement in the aggressive behavior of duodenal and small intestinal GISTs needs to be verified in vitro and in vivo, and further analysis is required, together with the accumulation of clinical samples of duodenal and small intestinal GISTs with trkB expression to determine its contribution to aggressive clinical outcomes.

By Nanostring analysis, genetic analysis of *NTRK2* in trkB positive GIST did not show amplification of *NTRK2* as a mechanism of trkB overexpression. On the other hand, significantly higher mRNA expression levels of *NTRK2* in trkB positive GISTs was observed when compared with those without trkB expression. These findings suggested possible transcriptional activation of *NTRK2* in this subset of GISTs, although *NTRK* fusion seemed to be less likely present.

In summary, trkB expression was found in duodenal GISTs and more than half of small intestinal GISTs, and seemed to be associated with poor prognosis. These findings could provide a possible hypothesis for trkB overexpression contributing to the aggressive clinical outcome in GISTs of duodenal origin.

### Supplementary Information

Supplementary Table 1.

Supplementary Table 2.

Supplementary Table 3.

Supplementary Table 4.

Supplementary Table 5.

Supplementary Table 6.

Supplementary Legends.

Supplementary Figure 1.

Supplementary Figure 2.

Supplementary Figure 3.

## Data Availability

The data of this study is available upon reasonable requests. Please contact to Tsuyoshi Saito (email: tysaitou@juntendo.ac.jp).
