# Supplementary material for: NTRK2 expression in gastrointestinal stromal tumors with a special emphasis on the clinicopathological and prognostic impacts
Source: Sci Rep. 2024 Jan 8;14:768. doi: 10.1038/s41598-024-51211-7 (PMC10774370; doi:10.1038/s41598-024-51211-7)
Supplement: Supplementary file 7 — Supplementary Legends. [file 41598_2024_51211_MOESM7_ESM.docx]

# Supplementary Figure 1: Blotto gram by nCounter Imbalanced assay for *NTRK2* (Red: GIST samples, black: Other tumors). Ratio of 5’-side expression/3’-side expression of *NTRK2* has been compared. None of the GIST cases appear outside the 95% prediction area consistently through multiple comparisons. Two samples consistently blotted outside the 95% prediction area are both invasive breast carcinoma samples (*NTRK2* fusion has not been detected by Archer analysis, however, other alterations need to be verified). Dashed lines imply 95% prediction lines.

# Supplementary Figure 2: Expression status of trkB ligands in GIST samples. There is no apparent difference of trkB ligands according to pan-trk/trkB expression. Several cases express trkB ligands at very low-level, but others do not express them at all.

**Supplementary Figure 3**: Differentially expressed top gene list according to pan-trk/trkB expression.
