# Supplementary figures and images for: NTRK2 expression in gastrointestinal stromal tumors with a special emphasis on the clinicopathological and prognostic impacts
Source: Sci Rep. 2024 Jan 8;14:768. doi: 10.1038/s41598-024-51211-7 (PMC10774370; doi:10.1038/s41598-024-51211-7)

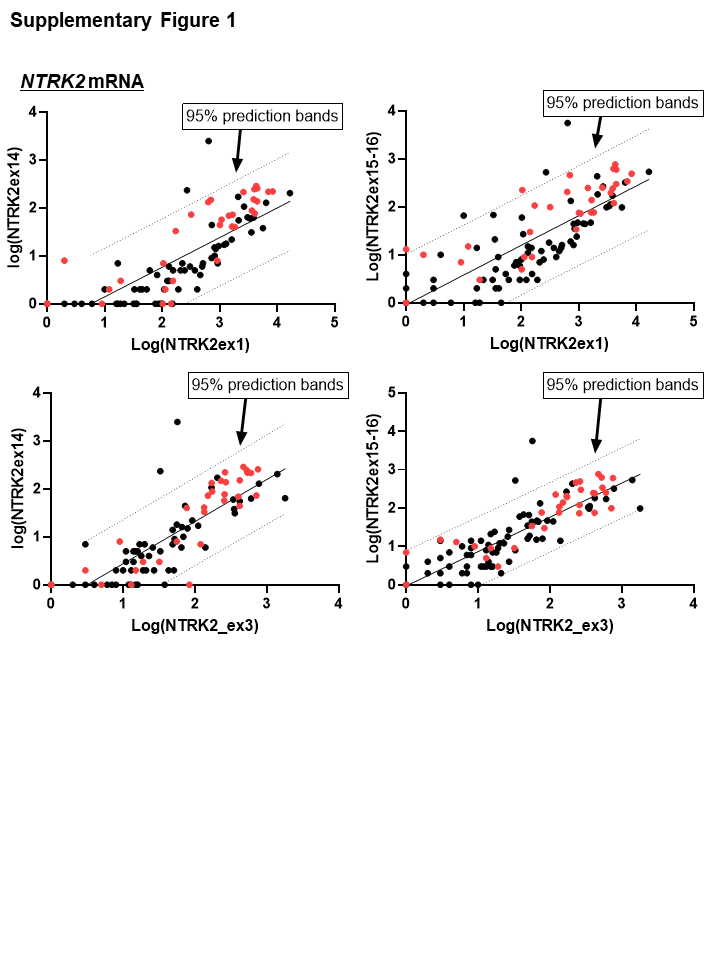

Supplement: Supplementary file 8 — Supplementary Figure 1. [file 41598_2024_51211_MOESM8_ESM.tif]

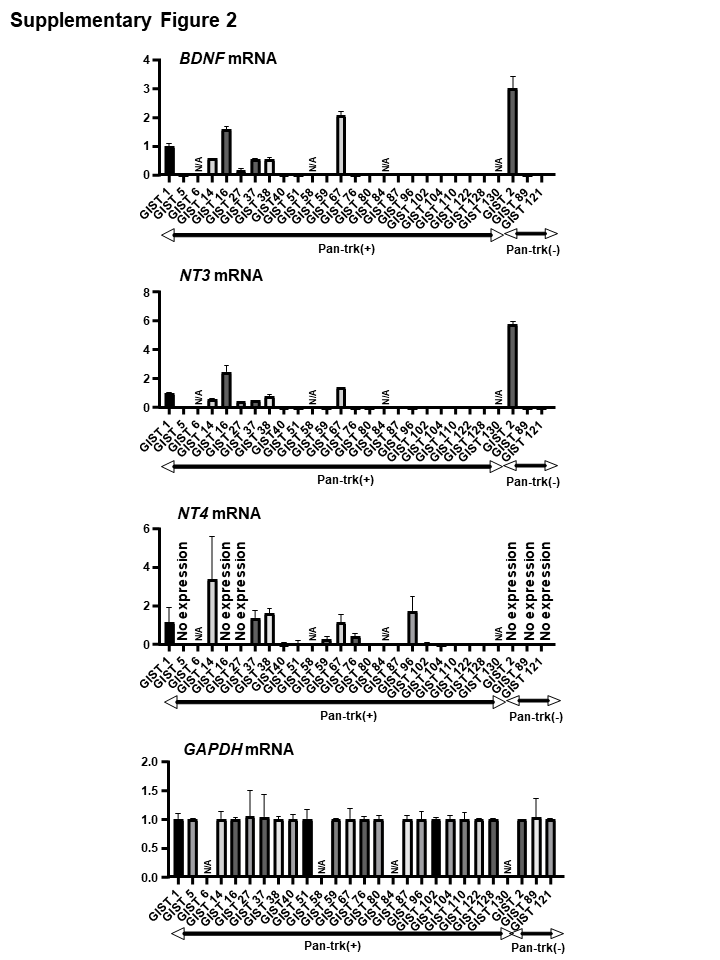

Supplement: Supplementary file 9 — Supplementary Figure 2. [file 41598_2024_51211_MOESM9_ESM.tif]

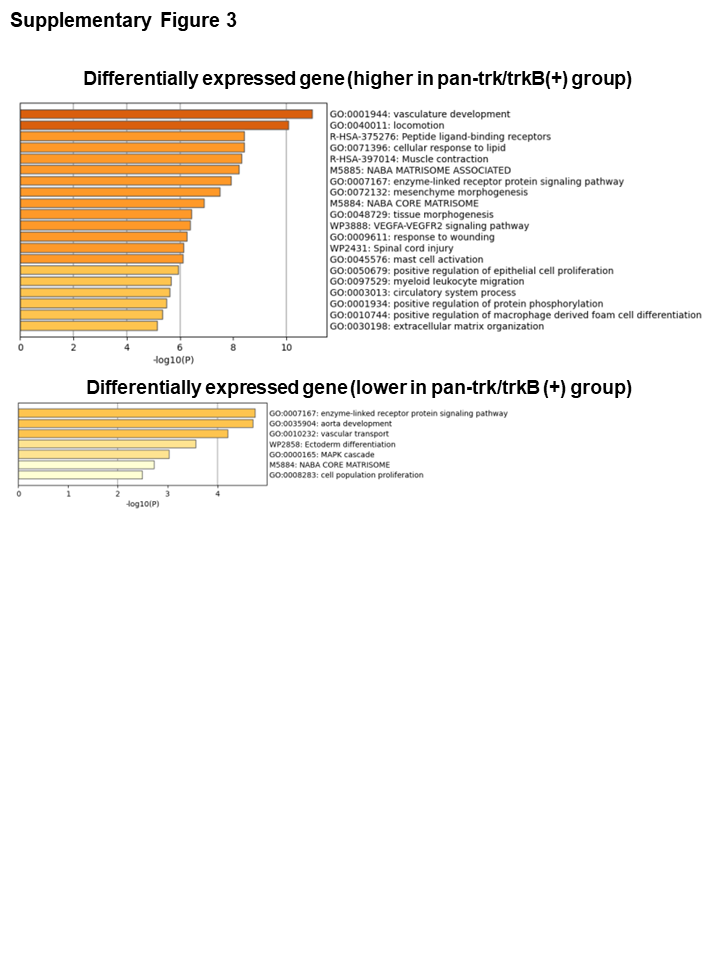

Supplement: Supplementary file 10 — Supplementary Figure 3. [file 41598_2024_51211_MOESM10_ESM.tif]
